# Apoptosis in *C. elegans*: lessons for cancer and immunity

**DOI:** 10.3389/fcimb.2013.00067

**Published:** 2013-10-18

**Authors:** Marios Arvanitis, De-Dong Li, Kiho Lee, Eleftherios Mylonakis

**Affiliations:** ^1^Department of Medicine, Division of Infectious Diseases, Rhode Island Hospital, Warren Alpert Medical School of Brown UniversityProvidence, RI, USA; ^2^School of Pharmacy, Second Military Medical UniversityShanghai, China

**Keywords:** *Caenorhabditis elegans*, apoptosis, cancer, immunity, cell death

## Introduction

Since 1974, when Sydney Brenner first introduced *Caenorhabditis elegans* in the scientific community as a model organism (Brenner, 1974), the nematode has been extensively used as a model system to study cellular biology. One of the most intriguing fields of molecular biology that have been investigated using this model host and that led to the 2002 Nobel Prize in Physiology or Medicine, is programmed cell death. Apoptosis is an evolutionarily conserved method employed by multicellular organisms to maintain tissue homeostasis during development and differentiation (Kuranaga, 2011), but can also serve as a way to prevent growth of cells mutated due to DNA damage (Bailly and Gartner, 2013).

## The main apoptotic pathway

In *C. elegans*, apoptosis is a normal component of growth. During development 1090 somatic cells are generated for each hermaphrodite, of which 131 invariantly undergo apoptosis (Sulston and Horvitz, 1977; Sulston et al., 1983). Interestingly, the main effectors of apoptosis in the worm are conserved in mammalian organisms. Indeed, the main apoptotic pathway in the nematode starts with the activation of EGL-1 in the cells that are destined to die. EGL-1 is a BH3 only protein which, when activated, binds to and inhibits CED-9, the only BCL-2-like protein in *C. elegans*, and thus negates its inhibiting effect on CED-4. CED-4 is the analog of mammalian APAF-1 and it serves as an activator of CED-3, a caspase, which then leads to cell death (Gartner et al., 2008).

Under-expression or mutation of effectors within the apoptotic pathway that are conserved in this model host has been known to lead to uncontrolled cellular proliferation and cancer in mammalian organisms. Indeed, *bcl-2* mutations are found in a wide range of human cancers (Ciardiello and Tortora, 2002). In support of this notion, a recent article reported an alternative mechanism of programmed cell-death activation on the nematode that involves inactivation of CED-9 by DRE-1. DRE-1 is the worm analog of the human protein FBXO10, which is known to be mutated or expressed at low levels in human diffuse large B-cell lymphomas (Chiorazzi et al., 2013).

Meanwhile, apoptosis seems to play an active role in *C. elegans* innate immunity as was shown by three pivotal studies. In the first one, Aballay et al. found that *Salmonella typhimurium* colonization of the *C. elegans* intestine leads to an increased level of cell death in the worm, dependent on the well-characterized EGL-1/CED-9/CED-4/CED-3 pathway (Aballay and Ausubel, 2001). In an ensuing article, the same researchers proved that *Salmonella*-induced apoptosis requires the *C. elegans* homolog of the mammalian p38 mitogen-activated protein kinase (MAPK) encoded by the *pmk-1* gene, a well-characterized and conserved innate immune effector (Aballay et al., 2003). Inactivation of *pmk-1* by RNAi blocked *Salmonella*-elicited *C. elegans* apoptosis, and epistasis analysis showed that CED-9 lies downstream of PMK-1 (Aballay et al., 2003). These results suggest that the apoptosis and immune response pathways are linked at some point to ensure the survival of the multicellular organism. A potential explanation for this link would be that apoptosis might be triggered by the host immune system to serve a protective role against the infectious process by eliminating infected cells thus hindering the dissemination of the invading pathogen. Interestingly, a similar relationship between immunity and apoptosis was recently shown in *Drosophila melanogaster* flies (Apidianakis et al., 2009). Specifically, the researchers investigated a *Pseudomonas aeruginosa* gut infection model in flies and found that the infection activated the c-Jun-N-terminal Kinase (JNK) pathway which in turn promoted apoptosis of infected enterocytes. Further, this phenomenon led to a subsequent over-proliferation of intestinal stem cells as a compensatory mechanism, thus suggesting a close interaction between immunity and pathways that control cell fate. In another article, researchers found that DAPK-1, the *C. elegans* ortholog of the tumor suppressor death-associated protein kinase, which is a known regulator of apoptosis and autophagy, decreases innate immune responses to barrier damage, thus protecting the worms from inflammation due to uncontrolled over activation of their immune system (Tong et al., 2009). This finding reveals a unique interplay between apoptosis, inflammation and cancer, suggesting that, throughout evolution, programmed cell death has acquired the role of protecting the organism from a wide variety of environmental insults.

## Apoptosis and DNA damage responses

The apoptotic pathway is directly linked to DNA damage control. To prevent growth of transformed cells, cell-cycle control proteins inhibit mitotic progression and promote apoptosis in response to DNA damage signals. These proteins are known as tumor suppression proteins and perhaps the most widely acknowledged among them is p53. Indeed, mutations in p53 have been found invariably in almost all different types of human cancer (Goh et al., 2011). The only p53-like protein in *C. elegans* is encoded by *cep-1* that is required for DNA-damage and UV-induced apoptosis. In the nematode, p53 is activated in response to DNA damage response signals and it induces *egl-1* and *ced-13* that encodes another BH3 only protein that serves in parallel to EGL-1 to promote apoptotic pathway initiation in such cases (Bailly and Gartner, 2013).

Interestingly, p53 is highly regulated in all organisms. In *C. elegans*, the main regulatory protein of p53 seems to be ATR, a serine/threonine protein kinase that recognizes single-stranded DNA generated by the recession of double-strand DNA breaks (Bailly and Gartner, 2013). However, recent studies have shown that there are other proteins that can regulate p53 either in parallel or together with ATR. For example, deletion of the gene encoding histone demethylase JMJD2, the human homologs of which are amplified in cases of cancer, slows DNA replication, blocks progression to S phase, and promotes ATR/p53-dependent apoptosis in the nematodes (Black et al., 2010). Further, a pivotal article elucidated the connection between Hypoxia-Inducible Factor 1 (HIF-1), a protein that is found to be upregulated in solid tumors and is associated with cancer prognosis, and apoptosis. The researchers used *C. elegans* to show that HIF-1 upregulates TYR-2, a member of the tyrosinase family in sensory neurons, which is then secreted and acts on the germline to antagonize *cep-1* dependent apoptosis (Sendoel et al., 2010). This observation not only identifies a potential adjunctive therapeutic target for tumors carrying the increased *hif-1* phenotype, but also shows that inhibition of apoptosis can sometimes be a non-autonomous cell response in multicellular organisms.

Importantly, an interesting interplay between pathways that are related to aging, cancer, and apoptosis was suggested by Perrin et al. (2013). The researchers investigated the interactions between DAF-2 (an insulin/IGF-1 homolog associated with aging), CEP-1, and AKT-1 (a protein that belongs to the Protein Kinase B/AKT family of protein kinases that are implicated in a wide range of human cancers). While AKT-1 inhibits CEP-1 and thus decreases DNA damage-induced apoptosis, DAF-2 antagonizes these effects and promotes apoptosis by parallel pathways through inhibition of AKT-2 and activation of *Ras* signaling. Therefore, the insulin/IGF receptors could serve as potential targets in AKT-dependent cancers.

Finally, other significant and recently identified effectors of the DNA damage response mechanism are the microRNAs (miRNAs), which have been shown to have an altered expression in tumor tissues and are implicated in the regulation of cellular response to radiation-induced DNA damage. Importantly, in a recent article, researchers investigated the role of miR-34, a conserved type of miRNA, on *C. elegans* and found that miR-34 has a differential effect in apoptotic vs. non-apoptotic cell death after radiation. In fact, miR-34 was shown to protect cells from non-apoptotic death while serving a role in promoting apoptosis (Kato et al., 2009). The significance of this finding becomes evident when one considers that the main response to radiation in some types of cancer is non-apoptotic death. Therefore, compounds that are able to lower miR-34 levels in these malignant cells could serve as important adjuncts to radiotherapy.

## Apoptotic corpse clearance

The final piece of the apoptotic machinery is the engulfment and degradation of the apoptotic corpse which is induced by certain signals expressed on the membrane of the dying cells. Notably, the most well-recognized of these signals, a protein known as phosphatidylserine, has been proven to serve a substantial role in protecting mammalian organisms from lung inflammatory disorders (Savill et al., 2003), thus establishing the role of this pathway as a method to prevent inappropriate immune activation due to accumulating dead cell remnants. In *C. elegans*, the engulfment is mediated by two pathways that include multiple proteins like CED-1, CED-2, CED-5, CED-6, CED-7, CED-12, and both converge at CED-10 (a Rac family GTPase) (Kinchen et al., 2005), while the degradation is mediated by signals involving the RAB-5 protein and ending with lysosomal degradation of the corpse (Conradt and Xue, 2005). Contributing to the well-appreciated notion that disturbance of the corpse degradation pathway can be related to various autoimmune disorders (Nagata et al., 2010), Haskin et al. described a molecular link between CED-1 and innate immunity in *C. elegans* (Haskins et al., 2008). They found that in *C. elegans*, CED-1 upregulates a family of genes encoding proteins with prion-like glutamine/asparagine (Q/N)-rich domains, known to be activated by ER stress and thought to aid in the unfolded protein response (Urano et al., 2002), thus rendering *ced-1* mutant worms immunocompromised and very susceptible to *Salmonella enterica* infection. These findings indicate that *ced-1* is required for the transcriptional activation of an unfolded protein response pathway essential for proper response to invading pathogens (Lamitina and Cherry, 2008). Despite the fact that the investigators suggested that the function of CED-1 in innate immunity is not dependent upon its function in apoptotic corpse engulfment, the importance of this observation cannot be overlooked as it implies that at least some of the effectors of the engulfment pathway can in fact have multiple roles, functioning to protect the organism against noxious stimuli resulting either from within the cell (in case of abnormally folded proteins) or from its surrounding environment (in the case of apoptotic corpse clearance).

On the other hand, the interaction between dying cell removal and cancer is less clear. In a recent article, researchers showed that *sli-1*, the homolog of the mammalian proto-oncogene *c-Cbl*, is able to inhibit engulfment of the dying cells through a previously unidentified pathway (Anderson et al., 2012). More importantly though, in another study, Suzuki et al. showed that XK-family proteins promote phosphatidylserine exposure on the membrane of dying cells in response to apoptotic signals and found that XK-Related Protein 8, a member of the XK family, is epigenetically repressed in some types of human cancer cells (Suzuki et al., 2013). Both of these findings suggest a mechanistic association between autoimmunity and cancer in which apoptotic corpse degradation seems to have a central role.

## Conclusions

**Figure 1:**
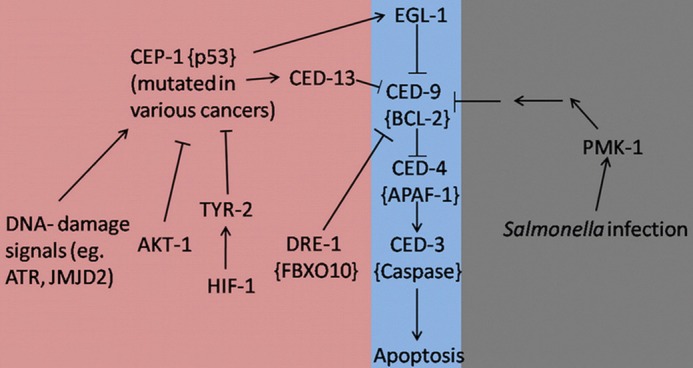
**Apoptosis signaling pathways in *C. elegans* during development (Purple), stress/infection (Gray), and hyperproliferation (Pink).** Lines with arrowheads represent activation while lines with barheads represent deactivation. Mammalian homologs are shown in brackets when applicable.

It is evident that research based on *C. elegans* has provided us with a wide variety of previously unrecognized interactions between programmed cell death and pathways that contribute to immunity or lead to cancer (Figure 1). It is now widely accepted that the apoptotic machinery serves a much wider role in multicellular organisms than what was previously acknowledged. It seems that this pathway is necessary to maintain tissue homeostasis not only under normal development but especially under conditions that are associated with cellular stress. In fact, apoptosis should be considered as the last physiologic safeguard in response to environmental insults. When all other repair mechanisms fail, programmed cell death is activated as a failsafe mechanism to sacrifice the affected cells for the greater good of the organism. Keeping this simple principle in mind, it is easy to deduce the interactions between apoptosis, cancer, and immunity. More specifically, infectious processes are well-recognized environmental insults, therefore programmed cell death can aid in preventing the dissemination of the pathogen especially when it comes to intracellular microbes. Similarly, in the case of cancer-inducing insults, like ionizing radiation, the apoptotic machinery is triggered in an effort to kill the malignant cells and protect the host from their uncontrolled proliferation that could be detrimental to its well-being. On the other hand, uncontrolled activation of programmed cell death can negatively impact the organism. Therefore, finding a way to specifically induce the apoptotic pathway in the affected cells could provide us with a powerful weapon in our fight against human diseases, like cancer and infectious processes.
